# Medical Cannabis for the Treatment of Migraine in Adults: A Review of the Evidence

**DOI:** 10.3389/fneur.2022.871187

**Published:** 2022-05-30

**Authors:** Babasola O. Okusanya, Breanne E. Lott, John Ehiri, Jean McClelland, Cecilia Rosales

**Affiliations:** ^1^Department of Health Promotion Sciences, Mel and Enid Zuckerman College of Public Health, University of Arizona, Tucson, AZ, United States; ^2^Health Sciences Library, University of Arizona, Tucson, AZ, United States; ^3^Division of Public Health Practice and Translational Research, Mel and Enid Zuckerman College of Public Health, University of Arizona, Phoenix, AZ, United States

**Keywords:** migraine, headaches, medical marijuana, medical cannabis, cannabinoids

## Abstract

**Background:**

Medical cannabis (MC) has been hypothesized as an alternative therapy for migraines, given the undesirable side effects of current migraine medications. The objective of this review was to assess the effectiveness and safety of MC in the treatment of migraine in adults.

**Methods:**

We searched PubMed, EMBASE, PsycINFO, CINAHL, and Web of Science for eligible studies in adults aged 18 years and older. Two reviewers independently screened studies for eligibility. A narrative synthesis of the included studies was conducted.

**Results:**

A total of 12 publications involving 1,980 participants in Italy and the United States of America were included.

Medical cannabis significantly reduced nausea and vomiting associated with migraine attacks after 6 months of use. Also, MC reduced the number of days of migraine after 30 days, and the frequency of migraine headaches per month. MC was 51% more effective in reducing migraines than non-cannabis products. Compared to amitriptyline, MC aborted migraine headaches in some (11.6%) users and reduced migraine frequency. While the use of MC for migraines was associated with the occurrence of medication overuse headaches (MOH), and the adverse events were mostly mild and occurred in 43.75% of patients who used oral cannabinoid preparations.

**Conclusions:**

There is promising evidence that MC may have a beneficial effect on the onset and duration of migraine headaches in adults. However, well-designed experimental studies that assess MC's effectiveness and safety for treating migraine in adults are needed to support this hypothesis.

## Highlights

- High-quality research on medical cannabis (MC) for migraine treatment is lacking
- MC reduced migraine headaches per month from 10.4 to 4.6 at follow-up (*p* < 0.0001)
- Medical cannabis aborted migraines in 11.6% of users
- Like amitriptyline, medical cannabis use resulted in reduced migraine frequency (~40%).

## Background

Migraine is a primary headache disorder and a clinical syndrome that is characterized by nausea and vomiting, photophobia, and phonophobia (1). Globally, migraine is a common disorder and the second leading cause of disability in both males and females younger than 50 years (2). It is estimated that migraine affects 1 billion people worldwide and 37 million people in the United States, with a prevalence of 20.7 and 9.7% in females and males, respectively (3).

Headache associated with migraine is often unilateral and pulsatile (3) and is described as migraine without aura (1). Migraine with aura is migraine headaches with a set of symptoms that result from cerebral dysfunction. The aura symptoms include visual, sensory, speech/language, motor, brainstem, and retinal symptoms (1), with symptoms usually preceding the headache by 20–30 min. Migraine is either episodic or chronic. Episodic migraines occur fewer than 15 days in a month, while chronic migraine is present ≥15 days per month (3). Chronic headaches have adverse effects on social relationships, job and may cause family distress (4).

The usual treatment modalities for migraine include paracetamol, non-steroidal anti-inflammatory drugs (NSAIDs), anti-emetics, and serotonin-receptor agonists -triptans (1, 3, 5). The effectiveness of these therapies varies due to individual patient differences, and they equally have adverse side effects that limit their duration of use (3, 5). To provide relief for patients with chronic migraine, surgical management in the form of peripheral nerve decompression has been reported with some success. Although 47% of patients who had surgery reported complete elimination of migraine, some people with chronic migraine did not have any benefits, despite the risks of surgical exposure (6, 7). This has led both physicians and patients to try dietary and herbal remedies, including the use of medical cannabis (MC) for migraine treatment (4, 8, 9).

Cannabis, or marijuana as it is commonly referred to, has been used for centuries to treat several ailments. Anecdotal client reports indicate benefits from marijuana or cannabidiol, a constituent of MC (3). The cannabis plant contains more than 100 cannabinoids, naturally occurring compounds, each with its own biological and medicinal properties. Two of these phytocannabinoids are Δ^9^- tetrahydrocannabinol (Δ^9^-THC) and cannabidiol (CBD) (3). While Δ^9^-THC has psychoactive properties, CBD does not (3). Instead, CBD causes sedation, which makes it more studied as a potential medical therapy (3). Marijuana is classified by the relative proportion of its two constituents as cannabidiol (high CBD: low Δ^9^-THC); intermediate (equal CBD: Δ^9^-THC); Δ^9^- tetrahydrocannabinol (low CBD: high Δ^9^-THC) (3).

Globally, disability due to migraine headaches is enormous, yet the commonly used medical treatments for episodic or chronic migraines have disturbing short- and long-term adverse effects (3). Although surgical intervention for migraine has been explored, the surgical risks and inconsistent patient outcomes make it a less than desired treatment option. A recent study investigating the corrugator muscle's resection with the trigeminal nerve's zygomaticotemporal branch's avulsion eliminated migraine headaches in 47% of participants while 6.6% of patients did not show any improvement after surgery (7).

Patient-reported relief of migraine symptoms (3) has fueled recent interest in the use of MC for migraines. In a survey of medical use of cannabis products in Germany, Austria, and Switzerland, 10.2% of patients with migraine reported self-use of cannabis (10). Also, 35% of respondents reported using MC for headaches/ migraine in another study (11). However, there is limited compelling evidence of its effectiveness in treating migraines. In addition to a lack of empirical evidence to support MC's safe use for migraine, there is some evidence that long-term or high-dose marijuana may pose health risks and exacerbate headaches. One such documented risk is reversible cerebral vasoconstriction syndrome with a high dosage of CBD, leading to ischemic or hemorrhagic stroke (3) and neuroinflammation of the meninges, causing diminished analgesia (4).

In the State of Arizona, United States, MC use is predominantly (94%) for chronic pain, including migraines, by MC cardholders (12). Given the increasing global interest in MC for the treatment of a wide range of ailments (13–15), and the proliferation of anecdotal information on the effect of cannabis on migraines in particular (3, 16, 17), it is important to summarize the evidence regarding the effectiveness and safety of MC for migraines. This systematic review's objective was to assess the effectiveness and safety of MC in the treatment of migraine in adults.

## Methods

We searched the following electronic databases from inception through November 19, 2020: PubMed; EMBASE; CINAHL; PsycINFO; and Web of Science, in consultation with a Medical Librarian (JM), for studies on medical marijuana and migraine in adults ≥18 years. An updated search of PubMed, EMBASE, CINAHL, and PsycINFO was performed on 26 January 2022. We included oral and inhalational routes of MC administration. There were no language restrictions during the search. Conference proceedings and abstracts returned in the systematic search were screened with the same eligibility criteria as peer-reviewed publications. The search strategies of the five databases are presented in Appendix 1.

Studies were eligible for inclusion if they compared MC with any treatment or “Nothing”. The eligible studies included randomized controlled trials, controlled “Before and After” studies, case-control studies, cohort studies, cross-sectional studies, case reports, or case series.

**Figure 1:**
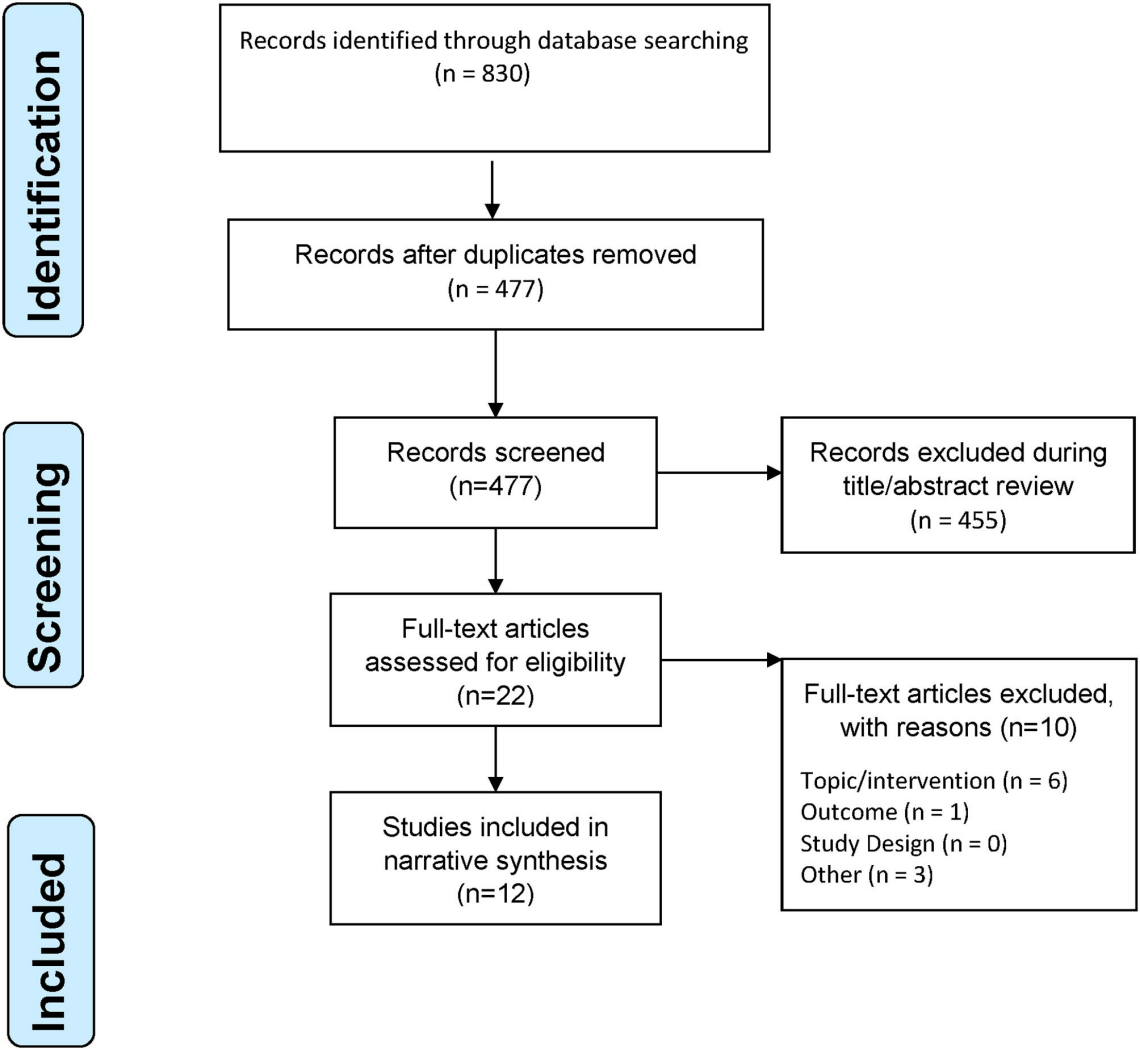
PRISMA flowchart of study selection process.

Two review authors (BO and BEL) independently screened the literature search results in two successive rounds of title and then title/abstract review to identify potentially relevant studies. Full reports of potentially relevant studies were obtained for further assessment. We resolved disagreements through consensus agreement within the review team and contacted the third author when there was a disagreement between two authors on study eligibility. One review author extracted data and checked it for accuracy by a second reviewer (BEL and BO). Data were extracted for the following variables: study design, sample size, location, population, treatment, control condition, outcomes, and findings. A narrative synthesis was used to describe the included studies because of the included studies' heterogeneity. The review was conducted following the Preferred Reporting Items for Systematic Reviews and Meta-Analysis (PRISMA) (18) (Figure 1). The review was exempt from ethics review, given that anonymized published data sources were used. However, this review protocol was registered (CRD42019129923) with the International Prospective Register of Systematic Reviews (PROSPERO) before starting the review process.

## Results

### Description of Included Studies

A total of 12 studies with 1,980 participants were included. The included studies were seven peer-reviewed publications (19–25) and five conference abstracts, including two case reports (26, 27), one case series (28), one retrospective chart review (29), and one randomized control trial (30). The seven peer-reviewed publications were retrospective cohort study (22), retrospective analysis of medical charts of migraine patients who used MC (19, 25) or analysis of MC Application (App) (20, 21) and online surveys (23, 24). The study selection process is shown in Figure 1. The included studies were presented at conferences or published from 2016 to 2021 and came from two countries, namely the United States (19–21, 23–25, 27, 29) and Italy (22, 26, 28, 30). The study characteristics are presented in Table 1.

**Table 1:** Study characteristics, population, treatment, outcomes, and findings of included studies.

**References**	**Design**	**Location**	**Source type**	**Population**	**Treatment and control**
Baraldi, et al. (22)	Retrospective cohort study	Italy	Peer-reviewed publication	32	Oral cannabinoid preparations at maximum daily dose of 1mL (25 drops) Control: None
Cuttler et al. (20)	Review of Archival data on Strainprint application (App)	U. S.	Peer-reviewed publication	653	Inhalational MC (smoking, vaping, concentrates, dab bubbler, dab portable) Control: None
Dini et al. (26)	Case report	Italy	Conference abstract	Male, 48 years old	Oral drop formulation with 6.5% THC and 8% CBD. Control: None
Gibson et al. (23)	Cross sections online survey	U.S. A	Peer-reviewed publication	161	Topical, Flower CBD, Edible CBD, concentrate CBD Control: None
Kesayan et al. (27)	Case report	U.S.	Conference abstract	Male, 38 years old	5 mg Dronabinol Control: None
Kuruvilla et al. (24)	Cross section (social media) survey	U.S. A	Peer-reviewed publication	164	Cannabinoid derivatives or cannabinoids Control: None
Lo Castro et al. (28)	Case series	Italy	Conference abstract	18	Cannabis oil (5-8% THC and 7-12% CBD) Control: None
Mechtler et al. (29)	Retrospective chart review	U.S.	Conference abstract	316	Not specified
Nicolodi et al. (30)	Randomized control trial	Italy	Conference abstract	79	Oral drop emulsion with 200 mg THC+CBD in a 200 ml 50% fat emulsion, daily for three months. Control: 25mg/day amitriptyline
Rhyne et al. (19)	Retrospective chart review	U.S.	Peer-reviewed publication	121	Vaporized, edible, topical, and smoked marijuana in varying doses and frequency. Control: None
Stith et al. (21)	Observational research using Releaf Application (App) data	U.S.	Peer-reviewed publication	284	Cannabis flower Control: None
Zhang et al. (25)	Retrospective electronic chart review	U.S. A	Peer-reviewed publication	150	Inhalation and orally ingested products Control: None

### Routes and Dosage of Administration

Migraine sufferers, ranging in age from 18 to 89, were treated with medical cannabis in various forms and doses (19). Oral drops of a THC and CBD formulation were administered in three studies (26, 28, 30). Oral cannabinoid preparations up to a maximum of 1 ml daily were used in a study (22), while topical, edible, and concentrate cannabinoids were used in two studies (23, 24). Both inhalational and orally ingested products were used by Zhang et al. (25) Dini et al. specified the formulation's composition as 6.5% THC and 8% CBD but did not specify dosage or treatment frequency (26). Also, Lo Castro specified 5–8% THC and 7–12% CBD in the MC oil in their study (28). Conversely, Nicolodi et al. reported a 200-mg daily oral dosing administered in a 200 ml 50% fat emulsion for 1 month but did not specify the THC/CBD composition (30). Only one patient received 5 mg dronabinol twice daily, without stating the route of administration (27). An included study neither specified the dosage nor the route of MC use (29). While two studies used inhalational routes to administer MC (20, 21); one with cannabis flower (21), another included study used a combination of oral, edible, and inhalational routes to administer MC products with a mean monthly dose of 2.64 ounces (vaporized), 2.59 ounces (edible), 2.73 ounces (topical), and 1.59 ounces (smoked) (19).

### Indications for MC

A total of three studies used daily MC for migraine prophylaxis (24, 26, 27). An included study described prophylactic use every day with an additional 200 mg dose permitted for acute pain treatment in the event of headaches (30). Participants of three other studies used MC for the treatment of migraines (22, 23, 25). In one study that assessed patients' self-reported reasons for MC use, 90.9% of patients reported using medical marijuana both preventively and for abortive migraine treatment (19).

### Relieving Effects of MC

The included studies measured migraine symptom relief with either symptom frequency (19, 26, 28–30) or migraine severity/intensity (20, 21, 27). The frequency was measured as the number of days with headache per month (26) or the number of headache events in a specified period of 1–3 months (19, 28–30). Migraine intensity was measured as perceived pain reduction using a Likert scale from 0 to 10 (27) or as the percent of pain relief (30). See Table 1 for included studies' characteristics.

Two included studies reported data on pain freedom (abortion of acute migraine headaches) (19, 28). Lo Castro reported a reduction of the number of days of migraine over 30 days of 0.86 (95% CI, 0.75–0.96) before and 0.75 (95% CI, 0.6–0.89) after MC use (*t*-test *p*-value = 0.2039) (28). In contrast, Rhyne reported a reduction in the number of migraine headaches per month; 10.4 at the initial visit and 4.6 at follow-up (*p* < 0.0001), and the abortion of migraine headaches in 14 users (11.6%) (19). MC significantly reduced nausea and vomiting associated with migraine attacks after 6 months of use (*p* = 0.0057) (22). Although MC did not significantly change the number of migraine days after the 6th month (*p* = 0.1182), it did change the acute medication consumption (*p* = 0.0006) and the number of days per month when at least one acute medication was used for migraine (*p* = 0.0004) when compared with baseline (22).

A study reported MC relieved migraines more than non-cannabis products (75.82% vs. 51.01%) (23). Also, in participants who relied on non-cannabis products and cannabis therapy, MC provided better relief (*p* < 0.001) (23), with sustained effect after controlling for migraine severity (*p* = 0.03) (23). In an online survey of MC users, there was an equivocal report of the usefulness of MC on headaches with 39% (64/164) patients reporting MC as not effective at all (no change in headache days), though 8.5% (14/164) reported MC to be very effective (50–100% reduction in headache days) (24).

The only included randomized control trial by Nicolodi et al. treated the control group with amitriptyline (30). In 79 migraine sufferers, THC+CBD treatment had a similar effect as amitriptyline (40.4% reduction in migraine attacks among the intervention group compared to a 40.1% reduction in migraine attacks in the control group) (30). Also, the use of 200 mg of cannabinoids as an adjuvant to patients on amitriptyline provided a further reduction of headache intensity to 43.5% among patients in the control arm (30).

In a 48-year-old patient with chronic migraine headaches, with previous pharmacotherapy treatment failure MC decreased headache frequency from >20 days to 0–1 day per month (26). Similarly, a 38–year-old had migraine pain intensity reduction, using a visual analog scale, from a 10/10 to 2/10 level following dronabinol treatment, and it remained effective for 3 years (27). In a case series of 18 patients, the number of days of analgesic use reduced from 1.83 (95% CI, 0.58- 3.07) at baseline to 0.85 (95% CI, 0.5–1.19) after 3 months (*t*-test *p*-value = 0.11889) (28).

Among 653 MC users, migraine severity was reduced in 87.3% of men and 88.6% of women (20), with a migraine rating reduction of 49.6% (*p* < 0.001) (20).

In a sub-group analysis of 284 (606 App sessions) MC users, Stith et al. reported symptom relief within 2 h (21). In another observational study involving 121 adult patients, the mean number of migraines per month decreased significantly from 10.4 at the initial visit to 4.6 per month at follow-up (*p* < 0.0001) (19). In the Rhyne et al. study, the positive (abortion of headaches) effects of MC were reported by 39.7% of patients, and negative effects were reported by 11.6% of patients (19). The positive effect of aborting migraine headaches was reported by patients vaporizing and smoking medical marijuana, probably due to the fast absorption associated with those methods of delivery (19). The negative effects reported included somnolence (1.7%, *n* = 2) and difficulty controlling the timing and intensity of the dose (1.7%, *n* = 2) with edible cannabis use (19).

### Adverse Events

The adverse events were mostly mild and occurred in 43.75% of patients who used oral cannabinoid preparations (22). Also, tolerance to the effect of MC indicated by a significant increase in dose across time per cannabis use session (*p* = 0.001) has been reported (19) and this might lead to the use of higher dosages of MC.

The use of MC for migraines has been associated with the occurrence of medication overuse headaches (MOH), compared with non-cannabis users (81 vs. 41%) (25). Also, current MC use for migraine is significantly associated with MOH [adjusted OR = 6.3 (CI 3.56–11.1)] (25). The relationship between MC use and MOH was further supported by a sensitivity analysis involving only people who developed MOH after commencing the MC use for migraines [OR = 2.6 (CI 1.52, 4.42)] (25).

## Discussion

There has been recent interest in using medical cannabis to treat many medical conditions, including migraines. The non-response of all patients to commonly used medical treatments (3), patient-reported relief of migraine symptoms with medical cannabis use, and biological plausibility for how MC may work to treat migraines have contributed to the increased interest. It is also difficult to predict which patients will benefit from surgical procedures for chronic migraine, with some patients showing no improvement post-operation (6, 7). As the number of states in the United States approving marijuana for medical use continues to increase, likely, migraine sufferers may choose cannabis to relieve their pain. Therefore, this review was conducted to assess the beneficial effects and safety of MC in treating migraine in adults.

After searching five databases for eligible studies, we applied very liberal criteria to include published abstracts and seven publications that reported the use of MC to treat migraines. The included publications were limited because they all used retrospective analysis of MC users' data in prescription charts or stored MC App data. Also, many of the clients had used or were current users of cannabis at enrollment, so no true pre-treatment condition existed in one of the publications (19). Despite this, all included publications showed some gradience toward symptom relief of migraine headaches with reduced frequency and severity. However, of concern is the reported onset of tolerance to, and medication overuse headaches from MC after prolonged use, as reported by Cuttler et al. and Zhang et al., respectively (20, 25). Since many people suffer migraine headaches for a long duration, there is a need for MC users and dispensaries to monitor the development of tolerance to MC.

This review's major limitation is the paucity of high-quality empirical studies on beneficial effects and safety of MC for the treatment of migraines. We did not find well-conducted published prospective research on MC and migraine in adults in the form of experimental studies. However, this review's strength is the comprehensive search of five major databases, which led to the identification of several conference abstracts and seven publications that were not included in a previous MC review for headache disorders (31). The abstracts were presented at the 2017–2019 conferences and included case reports and case series.

The evidence synthesized in this review demonstrates MC's potential for both prophylactic and abortive treatment of migraine. Cannabis use was associated with reduced headache frequency and pain intensity and had a similar effect to standard medical care. Still, the paucity of studies on migraine and medical cannabis and the lack of adequately powered studies that compared current treatment modalities of migraine with MC make it impossible to recommend MC to treat migraine with confidence. Also, possible side effects of marijuana such as medication overuse headache, and especially, the cerebral vasoconstriction syndrome that may lead to stroke (3), demonstrate the need for rigorous experimental studies to evaluate MC's effectiveness and safety for treating migraine in adults. Until more robust evidence exists on MC's effectiveness and safety for migraine, healthcare providers and patients should carefully weigh considerations for potential improvements in quality of life associated with pain freedom and reduced pain frequency with potential health risks of marijuana use for migraine in adults. Users of MC for migraines should also be aware of the potential to develop tolerance to MC, making them need higher MC doses over time.

To ascertain if MC is safe and effective in treating migraine in adults, future research should be experimental in design and enroll large numbers of participants. There is a need to investigate the best route of administration of MC to achieve an adequate serum level with minimal side effects. The optimal dosage of MC to treat adults with migraine also needs to be determined since low-dose cannabinoids might be ineffective, while a high dosage of cannabinoids might cause neuroinflammation, tolerance, and reduced analgesic effect (4). Equally, contemporary treatment modalities of migraine should be compared with MC to prevent migraines and for abortive emergency uses. There is a need to explore CBD-only therapy for migraine as the psychoactive THC component's exclusion might make some difference in treatment outcomes and pave new ways for both research and treatment with fewer legal restrictions.

## Conclusion

A review of three peer-reviewed publications and five gray literature sources (conference papers and posters) revealed that there is some evidence for MC's beneficial effect on treating migraine in adults. However, further research is needed to assess effective dosing and safety critically. Mindful of the upsurge of interest in MC use to treat migraines, there is an urgent need to implement well-designed studies to evaluate the effectiveness and safety of medical marijuana for treating adults with migraines.

## Data Availability Statement

The original contributions presented in the study are included in the article/Supplementary Materials, further inquiries can be directed to the corresponding author.

## Author Contributions

CR and JE conceived the topic. BO wrote the draft protocol, performed eligibility screening, and wrote the draft discussion of the review. BL revised the draft protocol, performed eligibility screening, performed data extraction, and revised the completed review. BL and BO drafted the results of the completed review. JM collaborated in developing the search strategy, performed the database search in March 2019 and an updated search in November 2020, and revised the draft review. JE and CR revised the draft protocol and completed the review. All authors agreed to the contents of the review for publication.

## Funding

This systematic review was funded through a grant entitled Research and Evaluation Services awarded by the Arizona Department of Health Services (ADHS) under Contract Number ADHS12-017291. The content of this publication is solely that of its authors and does not necessarily represent the official views of ADHS.

## Conflict of Interest

The authors declare that the research was conducted in the absence of any commercial or financial relationships that could be construed as a potential conflict of interest.

## Publisher's Note

All claims expressed in this article are solely those of the authors and do not necessarily represent those of their affiliated organizations, or those of the publisher, the editors and the reviewers. Any product that may be evaluated in this article, or claim that may be made by its manufacturer, is not guaranteed or endorsed by the publisher.

## Abbreviations

CBD: cannabidiol
MC: medical cannabis
MOH: Medication overuse headaches
TGV: trigemino-vascular system
THC: Δ^9^- tetrahydrocannabinol
PRISMA: Preferred Reporting Items for Systematic Reviews and Meta-Analyses
PROSPERO: Prospective Register of Systematic Reviews.
