# Supplementary material for: Medical Cannabis for the Treatment of Migraine in Adults: A Review of the Evidence
Source: Front Neurol. 2022 May 30;13:871187. doi: 10.3389/fneur.2022.871187 (PMC9197380; doi:10.3389/fneur.2022.871187)
Supplement: Supplementary file 1 [file Data_Sheet_1.docx]

**Appendix 1: Review search strategies**

| Database | Search date | Search strategy | Number of search outputs |
| --- | --- | --- | --- |
| PubMed | 1/26/2022 | (Headaches OR Headache OR "Headache"[Mesh]) AND ("Migraine Disorders"[Mesh] OR migrain* OR "hemicrania" OR "vascular headache" OR "vascular headaches") AND ("Delta-8-tetrahydrocannabinol" OR "therapeutic cannabis" OR "therapeutic marijuana" OR "CBD oil" OR THC OR CBD OR Nabilone OR dronabinol OR Tetrahydrocannabinol OR Cannabinols OR Cannabinol OR Cannabidiols OR Cannabidiol OR Cannabinoids OR Cannabinoid OR "Medicinal Marijuana" OR "Medicinal Cannabis" OR "Medical Cannabis" OR "Medical Marihuana" OR "Medical Marijuana" OR "Marijuana use"[Mesh] OR "Marijuana smoking"[Mesh] OR "Cannabis"[Mesh] OR "Cannabinoids"[Mesh] OR "Medical Marijuana"[Mesh] OR ("Cannabis"[Mesh] AND "phytotherapy"[Mesh]) OR (marijuana AND phytotherapy)) | 77 |
| Embase | 1/26/2022 | ('headache'/exp OR headache*) AND ('migraine'/exp OR migrain* OR 'vascular headache*' OR 'sick headache*' OR hemicrania) AND ('medical cannabis'/exp OR 'cannabinoid'/exp OR 'cannabis'/exp OR 'cannabis use'/exp OR 'cannabis smoking'/exp OR ('cannabis'/exp AND 'phytotherapy'/exp) OR (cannabis AND phytotherapy) OR (marijuana AND phytotherapy) OR 'medical marijuana' OR 'medical marihuana' OR 'medical cannabis' OR 'medicinal cannabis' OR 'medicinal marijuana' OR 'medicinal marihuana' OR 'treatment cannabis' OR 'treatment marijuana' OR Cannabinoid OR Cannabinoids OR Cannabidiol OR cannabidiols OR Cannabinol OR Cannabinols OR Tetrahydrocannabinol OR Dronabinol OR Nabilone OR CBD OR THC OR 'CBD oil' OR 'therapeutic marijuana' OR 'therapeutic cannabis' OR “Delta-8-tetrahydrocannabinol” OR 'delta8 tetrahydrocannabinol' OR 'tetrahydrocannabinol-cannabidiol combination') | 405 |
| CINAHL | 1/26/2022 | ((MH"Headache") OR headache) AND ((MH "Medical Marijuana") OR (MH "Cannabis") OR “Medical Marijuana” OR “Medical Marihuana” OR "Medical Cannabis” OR “Medicinal Cannabis” OR “Medicinal Marijuana” OR Cannabinoid OR Cannabidiol OR Cannabinol OR Tetrahydrocannabinol OR Dronabinol OR Nabilone OR CBD OR THC OR “CBD oil” OR “therapeutic marijuana” OR “therapeutic cannabis”) AND ((MH "Migraine") OR migrain* OR "sick headache" OR hemicrania OR "vascular headache" OR "vascular headaches") | 28 |
| PsycInfo | 1/26/2022 | (DE "Headache" OR headache) AND (DE "Migraine Headache" OR migrain* OR “sick headache” OR “sick headaches” OR “hemicrania” OR “vascular headache” OR “vascular headaches”) AND (DE "Marijuana" OR DE "Cannabis" OR DE "Marijuana Usage" OR “Medical Marijuana” OR “Medical Marihuana” OR “Medical Cannabis” OR “Medicinal Cannabis” OR “Medicinal Marijuana” OR "treatment cannabis" OR "treatment marijuana" OR Cannabinoid OR Cannabinoids OR Cannabidiol OR Cannabidiols OR Cannabinol OR Cannabinols OR Tetrahydrocannabinol OR Dronabinol OR Nabilone OR CBD OR THC OR “CBD oil” OR “therapeutic marijuana” OR “therapeutic cannabis” OR “Delta-8-tetrahydrocannabinol”) | 31 |
| Web of Science | 11/19/2020 | TS=headache AND (TS=migrain* OR TS="sick headache*" OR (TS=hemicrania OR TS="vascular headache*" ) AND TS=cannabis OR TS=((cannabis OR marijuana) AND phytother*) OR TS=medical marijuana OR TS=medical marihuana OR TS=Medical Cannabis OR TS=medicinal cannabis OR TS=medicinal marijuana OR TS=medicinal marihuana OR TS=cannabinoid OR TS=cannabidiol OR TS=cannabinol OR TS=Tetrahydrocannabinol OR TS=dronabinol OR TS=nabilone OR TS=CBD OR TS=THC OR TS=CBD oil OR TS=therapeutic marijuana OR TS=therapeutic cannabis OR TS=Delta-8-tetrahydrocannabinol OR TS=delta8 tetrahydrocannabinol) | 289 |
